# Cytisine—New Challenges of a Well-Known Drug in the Treatment of Nicotine Addiction

**DOI:** 10.3390/jcm15083146

**Published:** 2026-04-20

**Authors:** Lidia Bieniasz, Karol Wróblewski, Angelika Kamizela, Agnieszka Szyszkowska, Waldemar Grzegorzewski, Anna Czerniecka-Kubicka

**Affiliations:** 1 Department of Drug Form Technology and Medical Physics, Faculty of Medicine, Collegium Medicum, University of Rzeszów, 35-310 Rzeszów, Poland; lbieniasz@ur.edu.pl (L.B.); angelikakamizela@gmail.com (A.K.); szyszkowska.agnieszka@gmail.com (A.S.); 2 Laboratory of Physical Chemistry and Biophysics, Natural and Medical Center for Innovative Research, Faculty of Medicine, Collegium Medicum, University of Rzeszów, 35-310 Rzeszów, Poland; 3 Laboratory of Commercial and Non-Commercial Clinical Trials, Medical College of Rzeszow University, University of Rzeszow, 35-310 Rzeszow, Poland; kwroblewski@ur.edu.pl; 4 Laboratory of Metabolic Processes and Chromatographic Analyses, Natural and Medical Center for Innovative Research, Faculty of Medicine, Collegium Medicum, University of Rzeszów, 35-310 Rzeszów, Poland; 5 Faculty of Biology, Nature Conservation and Sustainable Development, University of Rzeszów, 35-310 Rzeszów, Poland; wgrzegorzewski@ur.edu.pl

**Keywords:** cytisine, nicotine addiction, pharmacotherapy

## Abstract

Cytisine is a plant-derived quinolizidine alkaloid found, among other sources, in the seeds of the common laburnum (*Laburnum anagyroides*). It has properties that enable it to act as a partial agonist of brain nicotinic α4β2 receptors, which play a key role in the development and maintenance of nicotine addiction. Clinical studies have shown that cytisine is a more effective smoking cessation aid than nicotine replacement therapy and at least as effective as varenicline in treating tobacco cigarette addiction. It may also be an effective agent in treating addiction to electronic cigarettes. Cytisine is also significantly cheaper than other anti-nicotine medications. This is of great importance for the population of smokers in developing countries, who cannot afford anti-nicotine treatment. In recent years, the role of cytisine in the pharmacotherapy of nicotine addiction worldwide has increased significantly. This drug is becoming available in an increasing number of countries, and in 2025 the World Health Organization (WHO) added cytisine to the list of essential medicines. The need for further development of the drug poses additional challenges for scientists, including the creation of new pharmaceutical forms, optimization of dosing regimens, and expansion of indications to include the treatment of nicotine addiction supplied into the body in forms other than traditional tobacco products. This review describes the use of cytisine in the treatment of nicotine addiction, the drug’s mechanism of action, pharmacokinetics, efficacy, safety of use, and the available pharmaceutical preparations. It also presents research directions on cytisine related to the development of innovative pharmaceutical products, new dosing regimens, and new indications associated with the treatment of addiction to various nicotine-containing products. Conclusions indicate that cytisine has a difficult dosing regimen, which is why patients do not adhere to it, limiting the effectiveness of the therapy. This necessitates optimizing the dosage of existing capsules and tablets or introducing, for example, new extended-release forms of the drug containing cytisine.

## 1. Introduction

Tobacco addiction is a complex and multi-factorial process with a global reach. It is mainly based on the desire to achieve the pharmacological effects of nicotine, as well as on learned associations; environmental factors also play a significant role in its development [1]. The tobacco epidemic is one of the biggest public health threats the world has ever faced, responsible for over 7 million deaths annually as well as disability and long-term suffering from tobacco-related diseases. Around 80% of the 1.3 billion tobacco users worldwide live in low- and middle-income countries, where the burden of tobacco-related illness and death is heaviest [2]. The most common causes of death among smokers are primarily cardiovascular diseases and cancers, such as lung cancer, cancers of the oral cavity and pharynx, larynx, kidney, pancreas, and stomach cancers [3].

The most effective method of reducing health risks and preventing mortality among smokers is to quit smoking. It has been proven that by stopping smoking, the risk of premature death associated with continued smoking decreases by about 90% [4]. Although the vast majority of smokers want to quit, achieving at least one year of nicotine abstinence without any aid is successful for only 5–7% of them [5]. At the same time, the Global Action to End Smoking study reports that approximately 1.2 billion people worldwide (including both men and women) use tobacco products, accounting for about 20% of the adult population [6].

In the treatment of nicotine addiction, it is recommended to use behavioral methods and pharmacological agents, among which nicotine replacement therapy (NRT), bupropion, and varenicline are recommended. All of these agents have fairly limited effectiveness, and the cost of a full course of treatment in many countries is often too high for most smokers and healthcare systems. As a result, pharmacological treatment of nicotine addiction is unavailable to millions of smokers worldwide. Therefore, one of the most important priorities in research on nicotine dependence treatment, and thus in the prevention of many life-threatening diseases, is the search for drugs that would not only be effective and safe but also accessible to smokers. Recent studies have shown that cytisine (CYT) is more effective than NRT and at least as effective, if not more so, as varenicline, which is considered the most efficient anti-nicotine drug [5]. Cytisine is currently also the cheapest agent treating nicotine addiction.

**Figure 1:**
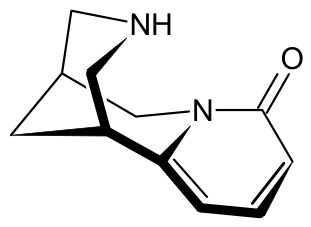
Chemical structure of cytisine.

CYT (Figure 1) is a herbal medicine that is currently available in the form of oral tablets or capsules, used in the treatment of nicotine addiction. Unfortunately, due to the inconvenient and complicated dosage of the preparation for patients, over 50% of smokers do not follow the treatment regimen, which significantly reduces the effectiveness of CYT medications [7]. Therefore, one of the current directions of research on cytisine is the optimization of dosing regimens.

This narrative review discusses the use of cytisine in the treatment of nicotine addiction, the mechanism of action of the anti-nicotine drug, pharmacokinetics, available pharmaceutical preparations, efficacy, and safety based on clinical study results. New directions for research on cytisine are also presented.

## 2. Cytisine History

The use of plants containing cytisine dates back thousands of years. Native Americans consumed parts of plants containing cytisine in magical rituals. In Europe, alcoholic extracts from Laburnum have been used in folk medicine for various purposes for hundreds of years. During World War II, the leaves of the Laburnum were used as a substitute for tobacco. Cytisine was first used in tablet form in Bulgaria in the 1960s as a medicine to help people quit smoking. A year later, the first clinical study was published showing the anti-nicotine effectiveness of alkaloids. Cytisine has been widely used in Central and Eastern Europe and Central Asia since the 1960s. Cytisine has been in use since 1964 and is now being registered in an increasing number of countries worldwide. Currently, cytisine is registered as a drug in 34 countries (mainly in Europe, Asia, and Africa). In many countries, including Poland and Canada, cytisine is available over the counter.

Cytisine was developed in the early 1950s by Professor Dimitar Paskov and Dr. Hristo Dobrev, but it was not widely used. Cytisine has been marketed under the trade name Tabex as a smoking cessation aid in many Central and Eastern European countries since the 1970s. However, in Poland, cytisine became widely used only in the late 1990s, thanks to the research of Professor Zatoński and his team, in collaboration with Professor West and other British scientists. Subsequent clinical studies conducted by various centers around the world confirmed the efficacy and safety of cytisine use. This allowed the WHO to include cytisine on the list of essential medicines in 2025 [8,9].

## 3. Mechanism of Cytisine Action

Cytisine is a naturally occurring alkaloid and has a wide range of biological activities [10,11]. From a pharmacological point of view, cytisine has similar effects to nicotine and varenicline. The difference between these effects is quantitative, not qualitative, though. Cytisine binds tightly to nicotinic receptors in the brain, but its impact on behavior, resulting from the stimulation of these receptors, is weaker than that of nicotine [10].

**Figure 2:**
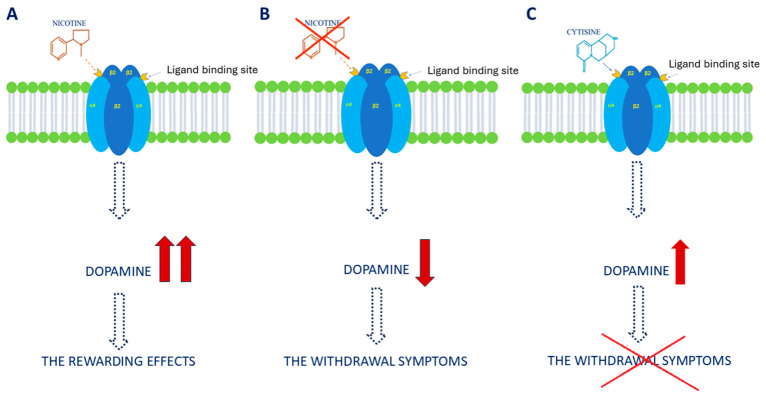
Mechanisms involved in nicotine addiction (**A**,**B**) and the therapeutic effects of CYT (**C**).

Cytisine, through N-cholinergic receptors, affects the central nervous system, the autonomic nervous system, and muscles. The alkaloid is a partial agonist of α4β2 receptor, primarily located in the CNS. This is the main receptor that cytisine binds to in the brain, causing an influx of Ca^2+^ and Na^+^ ions into the cell. After intravenous administration, the alkaloid reaches the highest concentrations in the thalamus, intermediate levels in the hippocampus, prefrontal cortex, and superior colliculi of the midbrain, which corresponds to the distribution of this receptor subtype in the CNS [12,13]. Its activation plays a key role in the development of nicotine dependence. Nicotine, upon crossing the blood–brain barrier, stimulates the limbic system to release neurotransmitters such as acetylcholine, noradrenaline, GABA, serotonin, endorphins, and the key neurotransmitter in terms of addiction mechanism, dopamine. The generation of a receptor protein response containing the β2 subunit by nicotine present in cigarette smoke stimulates dopaminergic signaling in the mesolimbic system, increases dopamine release in the nucleus accumbens and frontal cortex, and consequently leads to a rise in its brain levels, activation of the reward center, temporary mood improvement, and, with regular nicotine intake, addiction [14]. Due to its similar mechanism of action (please see Figure 2), cytisine competes with nicotine for the same receptors and gradually displaces it. By binding to the receptor site, it modifies dopamine release, reducing its concentration by approximately 50% compared to the amount stimulated by nicotine. In this way, it prevents full activation of the mesolimbic system, and through moderate stimulation of dopamine release, it alleviates the central symptoms of nicotine withdrawal. Simultaneous administration of both alkaloids reduces nicotine activity by 30% [15,16] and allows the gradual reduction of the body’s dependence on this substance, while avoiding withdrawal symptoms. Short-term use of cytisine leads to activation of nicotinic receptors, whereas long-term exposure to the alkaloid, even at lower concentrations than those required for stimulation, results in their desensitization [17,18]. Cytisine is also a partial agonist of α6β2 receptors, which are involved in the mechanism of dopamine release in the striatum and ventral tegmental area [19]. It binds to them with high affinity and produces an effect equal to 40% of maximum stimulation when acetylcholine is applied [20]. Cytisine also acts as a full agonist of homomeric α7 receptors—its affinity for them is Ki = 601, and the response generated by cytisine compared to the maximum effect observed for acetylcholine is 97% [21]. In addition to central effects, the alkaloid influences the autonomic nervous system, which is significant during nicotine addiction therapy [22]. The use of cytisine alleviates the somatic symptoms of withdrawal syndrome by activating pentameric α3β4 receptors, which are most densely concentrated in the autonomic ganglia and adrenal medulla. Its effect on the cardiovascular system is weaker than that of nicotine. No negative effects of the alkaloid on the autonomic nervous system are observed when doses recommended for nicotine addiction therapy are used [23,24]. Due to the abundant accumulation of α3β4 receptors in the Maynert bundle, their stimulation also modulates the mesolimbic dopamine pathway, which is the main reward center [25].

The central pharmacological action confirmed by numerous animal studies has shown that cytisine produces agonistic effects and relatively weak antagonistic effects in nicotine discrimination tests. Like nicotine, cytisine can act as a strong discriminative stimulus, although behavioral studies have shown that it is significantly weaker than nicotine [26]. Intracranial self-stimulation (ICSS) is a behavioral procedure used to assess the abuse potential of drugs. An elevated ICSS threshold reflects a dysphoric state, whereas a lowered threshold indicates enhanced reward function in the brain. Studies have shown that acute administration of nicotine and varenicline can lower ICSS thresholds. Acute administration of cytisine does not affect ICSS thresholds [27]. Discontinuation of chronic nicotine intake for 14 days increased in ICSS thresholds, which persisted for about 2 days. Varenicline and cytisine reduced the ICSS threshold increase induced by nicotine withdrawal. Overall, these studies suggest that cytisine may alleviate the dysphoric state associated with nicotine withdrawal, thereby protecting individuals from relapse.

The behavioral effects of intracerebral injections of cytisine in rats were studied [28]. Cytisine caused significant changes in behavior, whereas preliminary administration of nicotine did not alter subsequent responses to cytisine, suggesting that the effects of intracerebral cytisine injections are independent of behavioral sensitivity to nicotine observed after repeated exposure. It has been shown that repeated administration of cytisine to the brain has little effect on various behaviors. These results indicate that, unlike nicotine, cytisine does not induce rapid sensitization in locomotor functions.

## 4. Pharmacokinetics of Cytisine

The main pharmacokinetic properties of cytisine indicate that it is a highly hydrophilic active substance in its protonated form and lipophilic in its basic form [29]. Due to the protonation of the alkaloid in the acidic environment of the stomach and the resulting high polarity of the molecule [5], cytisine dissolves well in gastrointestinal fluids, but passes through biological membranes in the digestive tract less efficiently and crosses the blood–brain barrier less effectively than nicotine [30]. After oral administration, the alkaloid reaches peak plasma concentrations within 1–2 h [22,31]. Its half-life is 4.8 h, the residence time in the body is 6 h, and the volume of distribution is 110.1 ± 19.0 L. Cytisine is only slightly metabolized—the majority of the alkaloid is excreted unchanged by the kidneys [31,32]. Research shows that cytisine has an oral bioavailability of 90–95%, and food has no effect on the drug’s absorption in the digestive system [8].

Cytisine exhibits high bioavailability following oral administration; however, its penetration through cell membranes is limited by its physicochemical properties and degree of ionization. Cytisine has a basic pKa within the physiological range. This means that a significant proportion of the molecule exists in the protonated form at the pH of the gastrointestinal tract, which increases water solubility but limits passive diffusion through lipid membranes.

Due to its moderate lipophilicity (low log P/log D values in acidic and neutral environments), limited permeability of cyt through the cell membrane can be anticipated, which is consistent with theoretical models [30]. Compared with cytisine, nicotine is characterized by greater lipophilicity and a higher proportion of the non-ionic form at physiological pH, which enables more effective penetration of cell membranes and wider tissue distribution, including rapid penetration into the central nervous system. The bioavailability of ingested nicotine is reduced due to first-pass metabolism in the liver.

## 5. Cytisine Preparations and Dosing Regimen

The indication for the use of preparations containing CYT is the treatment of nicotine addiction. Currently, many OTC preparations whose active substance is this CYT are available on the pharmaceutical market: Tabex (coated tablets, Sopharma Warsaw Ltd., Warsaw, Poland), Desmoxan (tablets, hard capsules, Aflofarm Farmacja Polska Ltd., Tomaszów Mazowiecki, Poland), Cytisinum Aflofarm (hard capsules, Aflofarm Farmacja Polska Ltd.), Recigar (coated tablets, Adamed Pharma S.A., Piaseczno, Poland), Exicyt (coated tablets, Adamed Pharma S.A.), Cytisinicline APC Pharmlog (coated tablets, Adamed Pharma S.A.), Cytisinicline Pro-Pharma (coated tablets, Adamed Pharma S.A.), Punkamlar (coated tablets, Adamed Pharma S.A.), Denicit (coated tablets, Solinea Ltd., Warsaw, Poland), Nikozipix (coated tablets), Recigar Active (oral solution, Adamed Pharma S.A.), Cerdablan (oral solution, Adamed Pharma S.A.). All cytisine preparations are produced in a unit dose of 1.5 mg and are intended for 25 days of therapy. The oldest available product on the market is Tabex, produced since 1964 in Bulgaria (Sopharma) [33,34]. It can be purchased as an over-the-counter medicinal product in several countries in Europe and Central Asia (Poland, Lithuania, Latvia, Bulgaria, Belarus, Ukraine, Georgia, Armenia, Azerbaijan, Moldova, Serbia, Kazakhstan, Uzbekistan, Kyrgyzstan, Tajikistan, Turkmenistan) [34]. One package of the medicinal product containing 100 tablets allows for a full course of treatment. The dosage of cytisine preparations changes during the course of treatment (Table 1).

It is recommended that during the first three days of therapy, the patient takes 1 tablet (or capsule) every 2 h, a total of 6 per day. From day 4 to day 12—every 2.5 h, a total of 5 tablets per day; from day 13 to day 16 of the treatment—one dose every 3 h, a total of 4 tablets per day; on days 17–20, 1 tablet every 5 h, a total of 3 doses per day; and from days 21–25, 1–2 tablets per 24 h. The patient should completely stop smoking by the fifth day of the 25-day therapy. If the results of nicotine cessation treatment are unsatisfactory, it can be repeated after 2–3 months. In Canada, since 2017, cytisine has been available in capsule form (Cravv, 1.5 mg). The dosing regimen is the same as for medicinal products available on the Polish market [5]. It can be seen that dosing regimen may be difficult and inconvenient for many patients. Therefore, there is a need to search for new forms of cytisine administration that would enable the reduction of the number of doses administered per day while maintaining the same therapeutic effect.

Recently, an oral solution with cytisine (1.5 mg per dose) has been registered in Poland. One package of the medication (100 doses) is sufficient for a full course of therapy. The treatment duration is 25 days. The dosing regimen is analogous to that of solid forms of cytisine.

The current dosing regimen of cytisine contained in the summary of product characteristics is based on unknown foundations from the 1960s and, according to many experts, requires revision and optimization [35]. In recent years, clinical studies have been conducted in which a new dosing regimen was used. In the work by Walker et al. [36], a randomized clinical trial comparing cytisine with varenicline for the treatment of tobacco smoking addiction was described. In the group using cytisine, the standard 25-day dosing regimen was used, followed by a maintenance dose of 3 mg/day for up to 12 weeks. The study showed that cytisine was at least as effective as varenicline. In the work by Rigotti et al. [37], the efficacy and safety of cytisine in the treatment of tobacco cigarette addiction were demonstrated using a new dosing regimen. The drug was administered three times a day at a dose of 3 mg for 6 and 12 weeks. It was thus shown that prolonged dosing may be more effective than the previously used 25-day regimen.

## 6. Effectiveness and Safety of Cytisine

So far, many controlled and uncontrolled clinical studies have been conducted, confirming the effectiveness of cytisine in treating tobacco cigarette addiction (Table 2). One of the breakthroughs was the results of a clinical trial published in 2011 in the influential medical journal “New England Journal of Medicine.” The study showed that this substance was almost three and a half times more effective in helping people quit smoking compared to a placebo (8.4% vs. 2.4%; RR 6.0; 95% CI: 1.66–7.13; *p* = 0.001). The study included 740 participants who smoked at least 10 cigarettes per day. The cytisine product used in the study was Tabex, administered according to the standard 25-day dosing regimen. Further studies have shown higher effectiveness of cytisine compared to nicotine replacement therapy [37]. Clinical trials have also been conducted, indicating that cytisine is at least as effective as varenicline, a drug previously considered the most effective in treating nicotine addiction. So far, meta-analyses have been published on the use of cytisine in the treatment of nicotine addiction. In each case, the effectiveness of cytisine has been thoroughly demonstrated [38,39,40,41].

Published scientific research results indicate that treatment with cytisine is safe and free of serious adverse effects. Even when cytisine was administered at the highest daily dose (9 mg per day), many adverse effects were not observed, and those that did occur were of mild/moderate intensity and resolved without causing lasting or severe health consequences. Most commonly observed were gastrointestinal disorders such as nausea, vomiting, dry mouth, upper abdominal pain, taste changes, as well as sleep disturbances [37]. According to Rigotti et al., nausea, vivid dreams, and insomnia were reported in less than 10% of each group. Sixteen participants (2.9%) discontinued cytisine treatment due to adverse events. No serious adverse events related to the drug occurred [35]. Cytisine therapy may, however, be associated with slightly more frequent adverse events than NRT.

Cytisine represents a potential option for use in the pharmacotherapy of electronic cigarette addiction. However, to date, only one clinical study has been described evaluating the efficacy and safety of cytisine in adults addicted to nicotine-containing electronic cigarettes who wish to quit [35]. It was a multi-center, randomized, double-blind, placebo-controlled clinical trial in which treatment with cytisine (combined with behavioral support) was compared with placebo (with behavioral support). Participants (160 adults) were randomly assigned (in a 2:1 ratio) to receive cytisine at a dose of 3 mg three times daily (*n* = 107) or placebo (*n* = 53) for 12 weeks. The observation period was 16 weeks. Cytisine therapy was well tolerated and more effective compared to placebo, resulting in significantly more sustained abstinence during the last 4 weeks of treatment (31.8% vs. 15.1%). These results need to be confirmed in a larger study with a longer follow-up period.

## 7. New Challenges for Cytisine

Due to the complicated dosing regimen of available pharmaceutical preparations containing cytisine, research is being carried out on new formulations. Solutions are being sought that will increase patient comfort by allowing the dosing schedule to be simplified and prolonging the duration of the drug’s action, which in turn will facilitate therapy, improve patient adherence, and perhaps reduce the occurrence of side effects.

One of the promising drug candidates appears to be cytisine, contained in extended-release tablets absorbed sublingually. The tablets were prepared based on the methacrylic acid polymer, Eudragit^®^ RS100, which is characterized by low water solubility and pH-independent swelling, allowing proper control of active substance release. PEG1000 was used as a copolymer. Considering the possibility of using the preparation by patients with diabetes, xylitol was used as a sweetening agent instead of sucrose. The powders have been compressed into buccal tablets. This form of drug administration is convenient for the patient and helps avoid bioavailability issues associated with oral administration. Ex vivo studies show that cytisine in this form is water-soluble, stable in the conditions present in the oral cavity, has good permeability through the mucous membrane and cheek tissues, is absorbed directly into the circulatory system, and accumulates in the cheek tissue at about 500 µg/cm^2^. The active substance is fully released from the tablet through diffusion according to Fick’s laws, and the rate of the process can be controlled by using hydrophilic substances such as propylene glycol or xylitol, or polymers like PVP K90, in the appropriate proportion. Such promising research results suggest that buccal tablets could become a good alternative to the previously used forms of cytisine administration in the future [52,53].

In frame of proposal “Cytisine in the development pathway of a new prolonged-release medicinal product in nicotine addiction therapy” (no.: KPOD.07.07-IW.07-0142/24)., an innovative sustained-release formulation containing cytisine on a polymer carrier, poly(3-hydroxybutyrate) (PHB), has also been developed. However, further developmental studies are needed to create an appropriate pharmaceutical form, investigate pharmaceutical availability, and assess in vitro biocompatibility, pharmacokinetics, and in vivo biodistribution [54].

A new application form is also cytisine (0.1–10.0%) in the form of a liquid, mist, spray, or oral aerosol. The pharmaceutical composition also contains water, co-solvents such as glycerol and propylene glycol, a viscosity agent, a sweetening agent, and an inorganic acid, buffer, or salt, which maintains the pH of the liquid between 3.0 and 7.5. The preparation is sprayed directly onto the oral cavity and its mucous membrane using a bottle equipped with an atomizer. One dose contains 1.5 mg of cytisine. The proposed dosing regimen is similar to that used for tablets or capsules available on the market. The advantage of this form of alkaloid administration is that there is no need to take it with a specific amount of liquid, which increases patient comfort and allows for quick and discreet intake of the preparation, as well as reduces the frequency of gastrointestinal side effects. Another preparation containing cytisine, administered in the form of an oral spray composition, contains the alkaloid in an amount of 0.01 to 10% by weight, a hydrophilic nonionic emulsifier from the group of polymers or copolymers, alcohol, excipients, and water. The pH of the composition obtained in this way ranges from 7 to 10 [31].

In recent years, the number of people addicted to new nicotine-containing products such as electronic cigarettes and heated tobacco products has increased significantly worldwide. However, there is little research on the pharmacotherapy of addiction to these products. Recently, the results of the first study indicating the efficacy and safety of cytisine in the treatment of electronic cigarette addiction were published. The results are therefore consistent with the known effects of cytisine when used in people addicted to tobacco cigarettes, but they need to be confirmed in a study with a larger sample and a longer follow-up period. Nevertheless, cytisine is a promising agent for people wishing to quit using electronic cigarettes. It may therefore fill the existing gap in the pharmacotherapy of nicotine addiction. As mentioned earlier, recent studies on new dosing regimens of cytisine indicate better efficacy with extended use (e.g., 12 weeks) compared to the traditional 25-day regimen [36,42]. However, further clinical studies are also needed here to confirm the results obtained so far and to provide a basis for a potential change in the current dosing regimen in the summary of product characteristics for cytisine-containing medicinal products.

## 8. Methodology

This paper is a narrative review linking with structure and examining cytisine treatment regimens/adherence and affordability and cost-effectiveness together with new forms of cytisine drug. A literature search for the pre-clinical and clinical data, pharmacokinetics and new drugs of cytisine was conducted using electronic databases: Web of Science, PubMed, PsycINFO, Scopus, Cochrane Library, EBSCO, Medscape, ProQuest, Science Direct, Springer, and Wiley Online Library for data published until March 2026. The cytisine-specific search terms were ‘cytisine’, ‘nicotine receptor partial agonist’, ‘tabex’,’desmoxan’, ‘cytisus laburnum’ or nicotine addiction in the title or abstract or as keywords. The search terms did not include other names for cytisine, such as laburnine, baptitoxine, ulexine and sophorine.

## 9. Conclusions

Cytisine has been used for many years in the treatment of tobacco cigarette addiction. Its effectiveness and safety, documented in clinical studies, as well as the low cost of therapy, make cytisine an important tool in treating nicotine dependence, and its significance continues to grow. Despite its long history, this drug still shows significant development potential. The growing interest in cytisine is setting new directions for research. The first of these concerns the development of innovative pharmaceutical preparations, such as new oral forms of the drug or extended-release formulations. Such solutions may improve adherence to therapeutic recommendations and reduce the risk of relapses. Further improvement in the effectiveness of anti-nicotine therapies with cytisine can be achieved by introducing new dosing regimens that replace the traditional 25-day treatment with longer programs, for example, 12-week programs, similar to varenicline therapy. As current research shows, extending the use of cytisine can significantly improve the rate of sustained abstinence. The use of cytisine in treating addiction to other forms of nicotine delivery to the body becomes extremely important. In recent years, electronic cigarettes and heated tobacco products have become increasingly popular. Initial study results already confirm the effectiveness of cytisine in treating addiction to nicotine-containing electronic cigarettes. Further clinical studies are needed, including those that will also confirm the drug’s effectiveness in cases of addiction to other nicotine-containing products.

The development of innovative pharmaceutical formulations with cytisine, alternative dosing regimens, and research into new areas of application could make the drug a key component of modern pharmacotherapy for nicotine addiction worldwide.

## Figures and Tables

**Table 1:** Cytisine dosing regimen [5,8].

Treatment day	1–3	4–12	13–16	17–20	21–25
Time interval between doses of the medicinal product [h]	2	2.5	3	5	-
Daily dose in tablets	6	5	4	3	1–2

**Table 2:** Characteristics of clinical studies with cytisine.

No	Type of Study	Participants	Study Project	Interventions	Results	Reference
1.	Randomized, controlled, placebo, double-blind	adult smokers ≥10 cigarettes/day	cytisinicline 3 mg three times a day for 12 weeks; or 6 weeks of cytisinicline + 6 weeks of placebo; or placebo for 12 weeks.	All groups received behavioral support.	Both cytisinicline regimens were more effective than placebo in maintaining smoking abstinence.	Rigotti N. et al., 2025 [42]
2.	Cytisinicline in smoking cessation in a randomized, double-blind, controlled clinical trial (RCT)	810 adults who smoked cigarettes daily	In the ORCA-2 study, which included 3 groups, with a double-blind, placebo-controlled, randomized design, two treatment periods with cytisine (6 or 12 weeks) were compared with placebo, with an observation period of up to 24 weeks.	Participants were randomly assigned (1:1:1) to groups receiving cytisine at a dose of 3 mg three times daily for 12 weeks (*n* = 270); cytisine at a dose of 3 mg three times daily for 6 weeks, followed by placebo three times daily for 6 weeks (*n* = 269); or placebo three times daily for 12 weeks (*n* = 271).	Biochemically confirmed continuous abstinence from smoking during the last 4 weeks of treatment with cytisine compared to placebo (primary) and from the end of treatment up to 24 weeks (secondary).	Rigotti N. et al., 2023 [35]
3.	Varenicline versus cytisine in smoking cessation treatment in primary care Conditions: Randomized controlled trial	The study included 377 participants, over 18 years old, who smoked cigarettes and were motivated to quit the habit.	186 people were randomly assigned to the group treated with cytisine, and 191 to the group treated with varenicline.	• Varenicline—start 1 week before the planned quit date • Cytisine—according to the manufacturer’s standard dosing protocol	The primary endpoint for smoking cessation was self-reported 7-day abstinence at 24 weeks, while the primary endpoint for feasibility was adherence to the treatment plan.	Oreskovic T. et al., 2023 [43]
4.	Randomized, double-blind study Placebo-controlled study; parallel groups	The study included 132 participants over the age of 18 who smoked more than 10 cigarettes a day.	67 participants were randomly assigned to the group receiving cytisine, while 65 participants received a placebo	Participants were randomly assigned to receive cytisine or a placebo, along with five counseling sessions with a pharmacist; treatment was conducted according to the manufacturer’s standard dosing protocol (25 days).	The primary endpoint was the continuous abstinence rate (CAR) at week 48; CAR was also measured at weeks 2, 4, 12, and 24.	Phusahat et al., 2022 [44]
5.	Randomized controlled trial	869 participants in the lung screening study who smoked	Randomized controlled trial	• Behavioral support only • Behavioral support plus 40 days of cytisine (standard dose) • Behavioral support plus 84 days of cytisine (standard dose) The protocol included 3 psychological consultations for both groups: on the 7th, 14th, and 25th day from the start of cytisine therapy or from the screening at the beginning of the study.	CA in week 52—verified by CO ≤ 9 ppm	Pastorino U. et al., 2022 [45]
6.	A multicenter, double-blind, randomized, placebo-controlled phase 2b study of cytisine in adult smokers (ORCA-1 study)	254 participants were randomized	A double-blind, randomized, placebo-controlled clinical trial in which cytisinicline tablets or a placebo were administered along with behavioral support for 25 days	Each participant received treatment for 25 days. Participants were randomly assigned in a 2:2:1 ratio to groups receiving cytisine at a dose of 1.5 mg, cytisine at a dose of 3 mg, or placebo during 11 clinic visits [days 2, 3, 6, 12, 16, 20 during treatment, at the end of treatment (day 27 ± 2), and then weekly after treatment in weeks 5, 6, 7, and 8]. All participants received about 10 min of smoking cessation counseling during each clinic visit, conducted by experienced counselors.	The primary endpoint was the expected reduction in the number of cigarettes smoked by the end of treatment; secondary endpoints were biochemically confirmed 7-day abstinence at week 4 and continuous abstinence from weeks 5 to 8.	Nides M. et al., 2021 [46]
7.	Cytisine versus varenicline in smoking cessation: a randomized controlled trial	679 adult smokers	Open, parallel, randomized controlled trial	• Varenicline—2 × 1.5 mg/day (titrated dose in the first week). • Cytisine—days 1–3: 6 × 1.5 mg/day; days 4–12: 5 × 1.5 mg/day; days 13–16: 4 × 1.5 mg/day; days 17–20: 3 × 1.5 mg/day; and days 21–25: 2 × 1.5 mg/day. A maintenance dose of cytisine 2 × 1.5 mg/day was added from day 26 to week 12 to align it with the duration of varenicline treatment.	Primary: Cessation of smoking (CO) at 6 months (verified with exhaled CO concentration < 9 ppm) Secondary outcomes measured at 1, 3, 6, and 12 months from the quit date included: self-reported CO, 7-day PPA, CPD, time to (relapse) craving, adverse events, treatment adherence/compliance and acceptability, nicotine withdrawal/craving, and use of healthcare/health-related quality of life	Walker N. et al., 2021 [36]
8.	The effect of cytisine and varenicline on smoking cessation A randomized clinical trial	1452 adult smokers motivated to quit smoking	Non-inferiority study, randomized and open-label, using a single-blind method	• Cytisine 1.5 mg initially 6 times a day, then gradually reduced over 25 days • Varenicline tablets 0.5 mg, gradually increased to 1 mg twice a day No placebo control group.	Primary: 6-month continuous abstinence confirmed by a breath test for CO at the 7th month of follow-up. Secondary: self-reported continuous abstinence at the 3rd and 6th months; 7-day point prevalence abstinence 4 weeks after the baseline visit and at the 4th and 7th months of follow-up; cigarette smoking at each follow-up visit.	Courtnay R. et al., 2021 [47]
9.	Cytisine for smoking cessation in patients with tuberculosis: a multicenter, randomized, double-blind, placebo-controlled phase 3 trial	2472 adult smokers who were diagnosed with pulmonary tuberculosis in the past 4 weeks and who were motivated to quit smoking.	Randomized, double-blind, placebo-controlled trial	**•** Cytisine (9 mg on day 0, gradually reduced to 1.5 mg by day 25) **•** Placebo Randomization in the study groups was done in a 1:1 ratio. All participants received interactive, personalized behavioral support, which included a 5-min session on the registration day and a 10-min session on the quit day.	Primary: Continuous abstinence (CA) at 6 months, defined as self-report (not using >5 cigarettes, bidis, hookah, or smokeless tobacco products since the quit date). Abstinence was biochemically verified by exhaled CO levels < 10 ppm.	Dogar O. et al., 2020 [48]
10.	Cytisine versus nicotine in quitting smoking	1310 daily smokers calling the NZ National Quitline, aged 18+, motivated to quit smoking. Assigned to the group receiving cytisine (655) or to the group receiving open-label nicotine replacement therapy (NRT) (655)	Randomized controlled non-inferiority trial in parallel groups	• 25-day treatment with cytisine tablets (Tabex) + vouchers for NRT in case they are needed after completing the cytisine treatment • Standard care, i.e., an 8-week NRT treatment (patches, gums, or lozenges), tailored to the level of addiction, provided in the form of vouchers. All participants received standard Quitline support, i.e., on average 3 × 10–15 min phone calls over 8 weeks.	CAR self-assessment (5 cigarettes or fewer) after 1 month CAR and 7-day PPA (smoking cessation) after 1 week, 1 month, 2 months, and 6 months. Adverse events Validation: not used	Walker N. et al., 2014 [49]
11.	Placebo-controlled study of cytisine in smoking cessation	740 healthy adults	Single-center, double-blind, placebo-controlled, parallel-group, randomized study	Tabex tablets (1.5 mg cytisine): • first 3 days: 6 tablets per day • days 4–12: 5 tablets per day • days 13–16: 4 tablets per day • days 17–20: 3 tablets per day • days 21–22: 2 tablets per day • days 23–25: 1 tablet per day Placebo tablets, the same treatment regimen. The treatment period lasted 25 days. Counseling on quitting the habit, randomization, and prescription of medication during the initial visit; phone calls on the TQD day + 1 week later (+optional clinic visit). Clinic visit 4 weeks after TQD, followed by phone calls at 6 and 12 months, with a visit to confirm abstinence if it was recorded. Behavioral support was minimal to simulate the likelihood of actual conditions in countries where Tabex is available	Primary: abstinence confirmed by CO testing 12 months after the end of therapy. Abstinence is defined as smoking <5 cigarettes in the previous 6 months and no smoking in the week preceding the visit. Secondary outcomes: sustained abstinence confirmed by CO testing at 6 months of follow-up; 2-week PPA at 4 weeks; 7-day PPA at 12 months. Validation expired, CO < 10 ppm. Loss to follow-up: 79 (cytisine) and 89 (placebo) participants were lost to follow-up over 12 months. Rates of discontinuation or dose reduction were similar in both groups: 6.2% for cytisine and 4.6% for placebo. Other outcomes: adverse events, serious adverse events.	West R. et al., 2011 [50]
12.	A double-blind, randomized, placebo-controlled study of cytisine in smoking cessation among employees with a moderate level of addiction	197 adult smokers aged 20 and over, smoking at least 15 CPD, who had not previously used cytisine and were motivated to quit smoking. They were randomly assigned to the group receiving cytisine (100) or placebo (97). 26 individuals (15 receiving cytisine, 11 placebo) who did not take any medication were excluded from the study report.	Double-blind, placebo-controlled, randomized trial with parallel groups	Tabex tablets (1.5 mg cytisine): • first 3 days: 6 tablets per day; reduce smoking by half • days 4–12: 5 tablets per day; quit smoking completely • days 13–16: 4 tablets per day • days 17–20: 3 tablets per day • days 21–22: 2 tablets per day • days 23–25: 1 tablet per day Placebo tablets, same treatment schedule The treatment period lasted 25 days, with TQD on day 5. All participants received “behavioral counseling” (no further details)	Primary endpoint: CO-confirmed CAR from weeks 5 to 8 Secondary endpoint: CO-confirmed CAR from weeks 5 to 26 Validation was based on exhaled CO concentration ≤ 8 ppm Other endpoints: changes in health-related quality of life indicators, changes in body weight, adverse events and serious adverse events The percentage of patients by week 8 was 6 in the cytisine group and 7 in the placebo group; by week 26, 10 in the cytisine group and 16 in the placebo group	Vinnikov et al., 2008 [51]

## Data Availability

No new data were created or analyzed in this study. Data sharing is not applicable to this article.
